# Metabolic Reprogramming in CD8^+^ T Cells During Acute Viral Infections

**DOI:** 10.3389/fimmu.2020.01013

**Published:** 2020-05-22

**Authors:** Shubhranshu S. Gupta, Jin Wang, Min Chen

**Affiliations:** ^1^Department of Pathology and Immunology, Baylor College of Medicine, Houston, TX, United States; ^2^Interdepartmental Graduate Program in Translational Biology and Molecular Medicine, Baylor College of Medicine, Houston, TX, United States; ^3^Immunobiology and Transplant Science Center, Houston Methodist Research Institute, Houston, TX, United States; ^4^Department of Surgery, Weill Cornell Medical College, Cornell University, New York, NY, United States

**Keywords:** CD8^+^ T cells, metabolism, glycolysis, fatty acid oxidation, glutaminolysis, epigenetics, viral infections

## Abstract

CD8^+^ T cells represent one of the most versatile immune cells critical for clearing away viral infections. Due to their important role, CD8^+^ T cell activation and memory formation during viral infection have been the focus of several studies recently. Although CD8^+^ T cell activation and memory formation have been associated with metabolic alterations, the molecular understanding behind T cells choosing one type of metabolism over others based on their differentiation stage is still unclear. This review focuses on how the signaling molecules and cellular processes that are characteristic of CD8^+^ T cell activation and memory formation also play a critical role in selecting specific type of metabolism during viral infections. In addition, we will summarize the epigenetic factors regulating these metabolic alterations.

## Introduction

CD8^+^ T cells constitute one of the critical mediators of adaptive immune response. T cells are further classified into naïve, effector and memory T cells based on their state of differentiation. While naïve CD8^+^ T cells represent the population that has not been activated by cognate antigen and are yet to receive the activation signal, effector and memory CD8^+^ T cells are antigen experienced. In the context of viral infection, naïve CD8^+^ T cells are activated when antigen-presenting cells (APC) present cognate antigens (derived from viral peptides) in the context of MHC I along with CD4^+^ T cell help (1). After activation, these virus-specific CD8^+^ T cells eventually clear out the infection and enter a ‘contraction phase’ wherein majority of them undergo apoptosis (2). However, a small percentage of these activated CD8^+^ T cells survive and form memory CD8^+^ T cells. Activation of naïve CD8^+^ T cells results in a myriad of activation signals, which not only expand the pool of antigen-specific cells, but also reprogram them metabolically. Metabolic reprogramming is critical to ensure that the activated CD8^+^ T cells not only fulfill the increasing energy demand during clonal proliferation, but also generate the metabolic intermediates they require to synthesize cellular components for the daughter cells.

Among different metabolic pathways, aerobic glycolysis, glutaminolysis and oxidative phosphorylation are the most studied types in CD8^+^ T cells; as determined using several cellular metabolism assays including extracellular flux analysis, stable isotope labeling, liquid chromatography-mass spectrometry (LC-MS) and gene expression studies (3). Aerobic glycolysis, a cytosolic pathway, is characterized by conversion of glucose into pyruvate with net production of 2 ATP molecules per glucose molecule (4). Oxidative phosphorylation, a mitochondrial pathway, is characterized by transfer of electrons from electron donors (such as NADH and FADH_2_) to electron transport chain (ETC) that ultimately results in the generation of 2.5 ATP per NADH and 1.5 ATP per FADH_2_ molecule (5). Bulk of NADH and FADH_2_ entering ETC reaction in T cells are generated either during tricarboxylic acid (TCA) cycle or from fatty acid oxidation (4). Finally, glutaminolysis involves breakdown of glutamine into glutamate, followed by conversion of glutamate into anaplerotic substrate α-ketoglutarate (α-KG) that fuels cellular proliferation (6–8). While naïve CD8^+^ T cells depend predominantly on basal glycolysis and oxidative phosphorylation fueled by fatty acid oxidation to generate ATP for their survival (9–12), activated T cells upregulate aerobic glycolysis (10, 12–14) and glutaminolysis (15). However, when the activated effector CD8^+^ T cells differentiate into memory CD8^+^ T cells, metabolism is switched back to fatty acid oxidation fueled by long-chain and short/branched-chain fatty acids, as recently shown by us and others (16–18).

Progress in T cell immunology research in the past decade has led to significant advancement in our understanding about various molecular factors driving T cell activation and memory formation. However, it is still unclear as to how those molecular factors also determine metabolic fate in virus-specific CD8^+^ T cells. This review focuses on bridging that knowledge gap in CD8^+^ T cells during the course of anti-viral immune response.

## Brief Overview of Metabolic Regulation in Naïve CD8^+^ T Cells

Long term survival of naïve CD8^+^ T cells depends on continuous contact with self-peptide/MHC complex and IL-7 signaling (19). The expression of IL-7 receptor (IL-7R) is modulated by availability of IL-7 in T cell milieu. Depletion of IL-7 or loss-of-function mutation in IL-7R has been shown to severely restrict the survival of naïve T cells (20, 21), suggesting that signaling via IL-7R is critical for their long-term survival. Even though it is not clear if there is any cross-talk between signaling through self-peptide/MHC complex and IL-7 receptor (IL-7R), it has been proposed that signaling through self-peptide/MHC complex leads to the formation of lipid rafts which are then integrated into the cell membrane of naïve T cells; followed by recruitment of IL-7R to the cell surface by lipid rafts (19). In addition, expression of IL-7R in naïve CD8^+^ T cells can also be induced by FOXO1, a transcription factor recently identified to be responsible for quiescence in naïve T cells (22). Naïve CD8^+^ T cells, possessing a metabolically resting phenotype, rely primarily on fatty acid oxidation and basal glycolysis (9–13, 20) to meet their bioenergetic needs. Deletion of IL-7R leads to a reduction in glycolytic flux in naïve T cells (19, 20). This reduction in glycolysis is independent of any alteration in glucose uptake, since the expression of GLUT1 transporting glucose into T cells remained unchanged. Furthermore, IL-7R-mediated signaling also induces fatty acid oxidation-dependent oxidative phosphorylation in naive CD8^+^ T cells (23). Although the exact mechanism that underlies IL-7R-mediated modulation of basal glycolysis and oxidative phosphorylation still remains unclear, it's possible that upon activation of IL-7R, the resulting phosphorylation of STAT5 (pSTAT5) (20) and subsequent translocation of pSTAT5 to the nucleus causes it to activate transcription of glycolytic and fatty acid metabolism genes (24, 25). The dependence on both glycolysis and fatty acid oxidation could also be interconnected. The product of glycolysis, pyruvate, is converted into acetyl-CoA by pyruvate dehydrogenase (PDH), which can be then used to synthesize fatty acids. Fatty acids can further serve as electron donors in mitochondria during fatty acid oxidation (ß-oxidation) to generate ATP. Because naïve CD8^+^ T cells are resting in nature, their ATP needs are meant to largely sustain basal cellular processes.

IL-7 signaling-mediated activation of STAT1, STAT3, and STAT5 can further activate DNA and histone methyltransferases that methylate DNA and histone residues, respectively, in order to regulate gene expression (26–28). DNA methyltransferases (DNMTs) can methylate promoter regions associated with target genes and silence them; while histone methyltransferases (HMTs) can trimethylate lysine 4 on histone 3 (H3K4me3) or lysine 27 on histone 3 (H3K27me3) around target genes, resulting in gene activation or gene repression, respectively (29). So far, the metabolic targets of DNMTs and HMTs are largely unknown in naïve CD8^+^ T cells. Although DNMT such as DNMT1 has been reported to perform DNA methylation at *Ifng* locus in naive CD8^+^ T cells (30), whether it also methylates genes associated with naive CD8^+^ T cell metabolism is hitherto unknown. As far as HMTs are concerned, whether they differentially methylate H3K4 and H3K27 to maintain low levels of GLUT1 expression typical of naïve CD8^+^ T cells should also be examined. In addition, role of protein arginine N-methyltransferase 1 (PRMT1), an HMT which activates FOXO1 (31), could be tested in naive CD8^+^ T cells.

Taken together, although naive CD8^+^ T cells use IL-7 signaling to mediate basal glycolysis and oxidative phosphorylation, the genetic and epigenetic causes for their metabolic quiescence need to be further elucidated.

## Metabolic Reprogramming in CD8^+^ T Cells After Activation

Viral infection is typically followed by activation of innate immune system, where viral antigens are systemically taken up by antigen-presenting cells (APCs). Presentation of viral peptides in the context of MHC-I results in activation of naïve CD8^+^ T cells; although viral peptides could also be loaded onto MHC-II molecules in an autophagy- or endosome-dependent manner to activate naïve CD4^+^ T cells (32–35). Activation of naïve CD8^+^ T cells involves engagement of TCR by APCs across the immunological synapse. TCR engagement results in phosphorylation of immunoreceptor tyrosine-based activation motif (ITAM) by LCK, that further propagates TCR signaling and results in T cell stimulation. In addition to T cell stimulation, co-stimulation via CD28 eventually results in activation of AKT. Activated AKT induces nuclear translocation of NF-κB and expression of anti-apoptotic BCL-XL, resulting in T cell proliferation and production of IL-2 (36). AKT in conjunction with TCR signal activates mTOR (23, 24); which promotes T cell activation, proliferation and production of effector cytokines such as IFN-γ and TNF-α (37). mTOR activation further leads to expression of MYC and HIF1α (15, 38), which control expression of cell cycle progression genes (cyclin A, CDK2/4, cdc25a) (11) as well as activation markers and cytokines (including IFN- γ, TNF-α, TIM3, OX40, CD137, and Granzyme B) (39, 40). MYC and HIF1α can alternatively be induced by NFAT as well (41). NFAT is activated by calcium-dependent activation of calcineurin resulting from TCR signaling. Upon activation, NFAT translocates to the nucleus and induces the expression of T cell activation markers and cytokines such as CD40L, IL-2, and TNF-α; and promotes T cell proliferation via expression of cell cycle genes such as CDK4/6 and cyclin D1/D3 (42).

### Metabolic Regulation by T Cell Activation-Associated Molecular Factors

**Figure 1:**
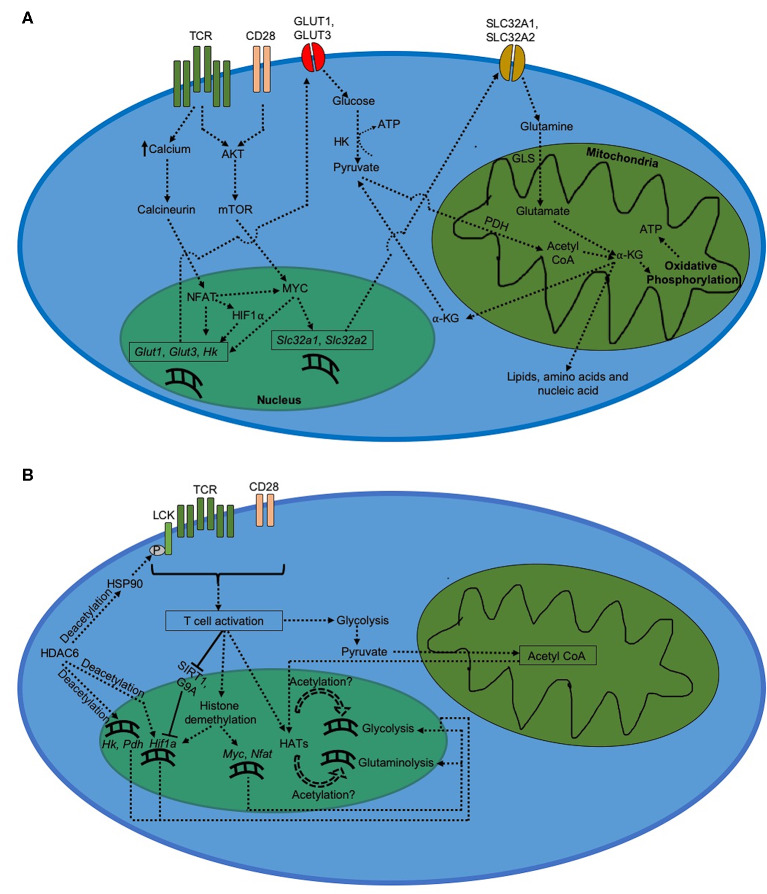
Metabolism in CD8^+^ T cells after activation. **(A)** Regulation of metabolism during CD8^+^ T cell activation. Activated CD8^+^ T cells demonstrate a drastic increase in glycolysis and glutaminolysis, which serve to generate ATP as well as biogenic precursors for daughter cells. **(B)** Epigenetic regulation of metabolism during CD8^+^ T cell activation.

Metabolism in activated CD8^+^ T cells is characterized by elevated level of glycolytic flux (Figure 1). The induction of glycolysis after TCR stimulation begins as soon as 15 min after TCR engagement (10). However, additional co-stimulatory signal via CD28 is required to keep glycolysis upregulated for longer duration (13, 14). This upregulation in glycolysis results from an increased expression of GLUT1 on the cell membrane due to AKT activation triggered by TCR and CD28 co-signaling (12, 14, 43). Increased expression of GLUT1 promotes glucose uptake into activated CD8^+^ T cells, which triggers glycolytic flux to produce pyruvate and synthesize ATP needed to meet increasing energy demands. Increased production of pyruvate during glycolysis is followed by its conversion into acetyl CoA, which enters TCA cycle to produce anaplerotic substrate α-KG required for the production of various cell components for new daughter cells (11, 44, 45).

MYC induced by AKT-dependent mTOR signaling can induce the upregulation of glycolysis in CD8^+^ T cells (15, 46, 47). MYC-dependent glycolytic regulation has been shown to be mediated through expression of miR-17~92 (48), a polycistronic microRNA cluster that increases the sensitivity of virus-specific CD8^+^ T cells to TCR stimulation (49). MYC also enhances glutaminolysis upon CD8^+^ T cell activation (11, 15). In addition, MYC increases the transcription of glutamine transporters, such as SLC32A1 and SLC32A2, required for the uptake of glutamine into activated T cells (50). This is followed by increased glutaminolysis, where glutamine is catabolized into glutamate by glutaminase (GLS). Glutamate is then converted into α-KG via the TCA cycle, which eventually results in biosynthesis of lipids, nucleotides and proteins necessary for T cell proliferation (45, 51). Via TCA cycle, α-KG further generates NADH and FADH_2_ that undergo oxidative phosphorylation to produce ATP in mitochondria. In addition, pyruvate formed during glycolysis can enter mitochondria and generate acetyl CoA with help of pyruvate dehydrogenase (PDH) (45).

During CD8^+^ T cell activation, glycolysis is also activated by NFAT via increased expression of GLUT1 and GLUT3 (41). In addition, NFAT upregulates expression of hexokinase (HK), which is a rate-determining factor in glycolysis in eukaryotes (41, 52). Increase in NFAT activation further increases expression of MYC and HIF1α in CD8^+^ T cells (53). Increased HIF1α-mediated glycolysis in activated CD8^+^ T cells (10) further suggests that NFAT plays a critical role in the expression of glycolytic activators during CD8^+^ T cell activation (Figure 1A).

Although the critical roles of glucose and glutamine uptake have been elucidated in regulating glycolysis and glutaminolysis, respectively, it is still unknown whether these metabolic pathways are interdependent in activated CD8^+^ T cells during acute viral infections. In fact, in activated alloreactive T cells, glutamine has been shown to undergo gluconeogenesis (54), a pathway characterized by the synthesis of glucose from non-sugar precursors. Hence, it is possible that glucose, which virus-specific activated CD8^+^ T cells acquire via GLUT1- and GLUT3-mediated uptake, could also be generated from glutamine via gluconeogenesis. This gap in knowledge, however, remains to be addressed.

### Epigenetic Regulation of T Cell Activation and Metabolism

The switch in metabolism from a quiescent state in naïve CD8^+^ T cells to an activated state after activation is accompanied by significant increase in cellular respiration, which results from a rapid upregulation of glycolysis and glutaminolysis. Changes in epigenetic regulation of metabolic genes could be responsible for this outcome. Increased expression of genes depends upon how accessible they are to cellular transcription factors. Eukaryotic cells typically make genes more accessible by loosening the chromatin-histone interaction around the target genes, which occurs by modifications at the histone sites. Gcn5, a histone acetyltransferase (HAT) induced by TCR signaling, has been shown to be recruited by NFAT to *Il-2* promoter and facilitate T cell activation via H3K9 acetylation (55). However, in order to do so, HATs require acetyl groups whose availability depends on acetyl CoA levels (56). Acetyl CoA is formed from pyruvate which is an end product of glycolysis. An initial upregulation in glycolysis due to TCR signaling feeds more acetyl CoA to HATs, resulting in glycolytic and glutaminolytic genes becoming more accessible to transcription factors like MYC, NFAT, and HIF1α. This could further increase glycolysis and glutaminolysis after CD8^+^ T cell activation. In addition, several studies have shown critical roles played by different subtypes of histone deacetylases (HDAC) in regulating CD8^+^ T cell activation and metabolism. HDAC6, a cytoplasmic isoform of HDAC (57), has been shown to deacetylate heat shock protein 90 (HSP90) resulting in LCK phosphorylation upon TCR engagement and activation of CD8^+^ T cells (58). Inhibition of HDAC6 reduces expression of critical glycolytic genes such as HK, PDH, and HIF1α, thereby impairing glycolytic flux during activation of lymphocytes (59). Furthermore, HDAC5 has also been shown to mediate optimal IFN-γ production during CD8^+^ T cell activation (60). On the other hand, HDAC2 represses optimal IL-2 production during CD8^+^ T cell activation via histone H3 deacetylation; and hence CD8^+^ T cells downregulate HDAC2 activity at *Il2ra* locus early during the activation in order to sustain optimal IL-2 production during anti-viral primary response (61). Likewise, SIRT1, a class III HDAC whose expression is inhibited by IL-2 signaling, also negatively regulates CD8^+^ T cell activation via deacetylation of *Hif1a*; leading to downregulation of SIRT1 expression by activated T cells to sustain optimal glycolysis and IFN-γ production (62–66).

Differential methylation of histone proteins associated with target genes has also been reported to play critical role in regulating gene expression during CD8^+^ T cell activation (Figure 1B). For instance, histone demethylation remodels chromatin into an open conformation. After viral infection, *Myc, Nfat* and *Hif1a* genes get increasingly demethylated upon activation of naïve CD8^+^ T cells (67). DNA methyltransferases (DNMTs) can also silence gene expression via methylation of cytosine residues of CpG dinucleotides in the promoter region. During viral infection, CD8^+^ T cells upregulate DNMT3a expression which ultimately leads to silencing of *Tcf7* gene in virus-specific activated CD8^+^ T cells (68). Absence of TCF7 in activated CD8^+^ T cells has been reported to downregulate *Hif1a* expression (69), suggesting that preventing DNMT3a-dependent *Tcf7* silencing during T cell activation could potentially improve HIF1α-mediated glycolysis in virus-specific CD8^+^ T cells. This hypothesis, however, needs to be tested under experimental setting. In addition, histone methyltransferases (HMTs) can methylate histones associated with genes involved in T cell activation. For example, differential methylation at lysine 9 site on histone H3 (H3K9 methylation) by G9a leads to repressive chromatin conformation around *Hif1a* gene (70, 71). Because of its inhibitory role in regulating T cell activation, virus-specific CD8^+^ T cells restrict G9a activity at *Il2ra* locus early during activation (61, 70, 71). Furthermore, Enhancer of Zeste Homolog 2 (EZH2), which represses gene expression via H3K27 trimethylation, is induced upon T cell activation and is critical for repression of FOXO1, an inhibitor of glycolysis (28, 72), in virus-specific activated CD8^+^ T cells (73).

Despite the availability of plethora of information about epigenetic regulators of metabolism in virus-specific activated CD8^+^ T cells, several questions remain unanswered. For instance, although it is known that *Myc, Nfat*, and *Hif1a* genes are increasingly demethylated during CD8^+^ T cell activation (67), the epigenetic mechanism underlying that demethylation process is still unclear. Either DNA demethylases or histone demethylases (or both) may play a role here, but the specifics around it are still lacking. Moreover, whether preventing DNMT3a-dependent *Tcf7* silencing during CD8^+^ T cell activation could potentially improve HIF1α-mediated glycolysis also needs to be experimentally demonstrated. In addition, the epigenetic regulation underlying MYC-driven glutaminolysis in virus-specific activated CD8^+^ T cells also remains to be addressed.

## Metabolic Reprogramming During CD8^+^ T Cell Memory Formation

During CD8^+^ T cell primary response, viral infection is eventually cleared off. This is followed by most of the virus-specific CD8^+^ T cells undergoing programmed cell death, a phase termed as contraction phase (2). IL-15 signaling plays a critical role in controlling the survival of virus-specific activated effector CD8^+^ T cells through the contraction phase (74, 75) via induction of *Nix* and *Runx2* expression (18, 76); thereby regulating the formation of T cell memory against viral infections (77). The indispensable role of IL-15 signaling is further demonstrated by its ability to induce p70 S6 kinase-mediated homeostatic proliferation in virus-specific memory CD8^+^ T cells; and prime them to rapidly enter cell proliferation upon future viral re-infection (78). In addition to IL-15 signaling, anti-viral T cell memory formation is also regulated by autophagy, a cellular recycling process wherein cells break down their own components via autophagolysosomal fusion (79, 80). The requirement of autophagy during T cell memory formation is linked to its role in clearing off dysfunctional mitochondria in virus-specific CD8^+^ T cells (18, 80). In this section we will discuss the mechanisms through which IL-15 signaling and autophagy regulate metabolism during anti-viral CD8^+^ T cell memory formation.

### Regulation of Long-Chain Fatty Acid Metabolism by T Cell Memory-Associated Molecular Factors

During the contraction phase, the surviving cells that would eventually form memory CD8^+^ T cells are characterized by gradual transitioning to a more quiescent metabolic phenotype from an activated metabolism. Despite their metabolic quiescence, virus-specific memory CD8^+^ T cells are metabolically more fit compared to activated effector CD8^+^ T cells, as demonstrated by higher expression of metabolic fitness-associated genes (81). Memory CD8^+^ T cells use long-chain fatty acid oxidation instead of glycolysis (16, 43). This reduced dependence on glycolysis during T cell memory formation is due to increased expression of FOXO1 (31, 72, 82). On the other hand, dependence on fatty acid oxidation during formation of CD8^+^ T cell memory is mediated by both TRAF6, a negative regulator of T cell activation, as well as IL-15 signaling (16, 83, 84). During memory formation, IL-15 is either trans-presented by APCs (85, 86) or produced by CD8^+^ T cells themselves (87). IL-15 signaling activates mitochondrial biogenesis mediated by transcription factor A, mitochondrial (TFAM); thereby increasing mitochondrial density in CD8^+^ T cells (83). We recently showed that the quality of these mitochondria during CD8^+^ T cell memory formation is regulated by NIX, a mitochondrial outer membrane protein, which is induced by IL-15 signaling during the contraction phase (18). In addition, IL-15 signaling during CD8^+^ T cell memory formation has been shown to increase expression of CPT1A, a metabolic enzyme that transports long-chain fatty acids into mitochondria thereby mediating fatty acid oxidation (83). We showed that this IL-15-dependent induction of long-chain fatty acid oxidation in virus-specific memory CD8^+^ T cells is regulated by NIX via prevention of HIF1α accumulation during the contraction phase (18). Long-chain fatty acid oxidation plays a critical role during anti-viral T cell memory formation by maintaining optimal ATP levels necessary for memory precursor effector CD8^+^ T cells (MPECs) to evade apoptosis during the contraction phase, leading to successful differentiation into memory CD8^+^ T cells (18).

**Figure 2:**
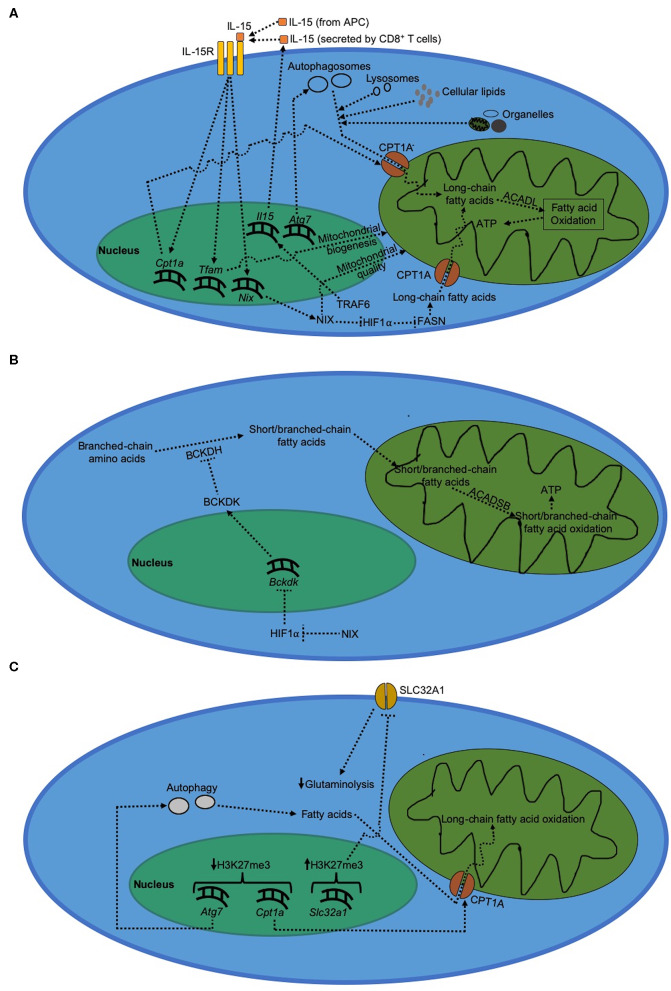
Metabolism in CD8^+^ T cells during memory formation. **(A)** Regulation of long-chain fatty acid metabolism during CD8^+^ T cell during memory formation. Immunological memory formation is characterized by metabolic shift to fatty acid oxidation driven by long-chain fatty acids, which serves to primarily generate ATP. **(B)** Regulation of short/branched-chain fatty acid metabolism during CD8^+^ T cell memory formation. **(C)** Epigenetic regulation of metabolism during CD8^+^ T cell memory formation.

Long-chain fatty acid metabolism by T cell memory-associated molecular factors is critical for memory T cell formation (Figure 2A). The increased demand for long-chain fatty acids during CD8^+^ T cell memory formation is met through lysosomal lipolysis (17). Although it is not clear as to what triggers lysosomal lipolysis during memory formation, one possible pathway could be autophagy. Consistent with this possibility, Cai et al. (88) showed that mitochondria receive long-chain fatty acids for fatty acid oxidation from the phospholipids degraded via autophagy. Hepatocytes have also been shown to use autophagy to deliver cellular lipids to lysosomes and break them down into free long-chain fatty acids in order to carry out mitochondrial fatty acid oxidation (89). As to where does the cellular lipids come from within CD8^+^ T cells during memory formation, O'Sullivan et al. (17) showed that CD8^+^ T cells can synthesize their own lipids using fatty acid synthase (FASN). Besides cellular lipid biosynthesis, another source of fatty acids is active degradation of cell organelles themselves via autophagy. Mammalian cells degrade dysfunctional mitochondria via mitochondrial autophagy (mitophagy) that is induced by NIX in virus-specific CD8^+^ T cells to fuel long-chain fatty acid oxidation inside healthy mitochondria (18, 90). Moreover, we and others have also shown that absence of mitochondrial autophagy during the contraction phase impairs the ability of CD8^+^ T cells to actively degrade dysfunctional mitochondria, thereby leading to a defective memory formation and long-chain fatty acid oxidation (18, 79, 80). In addition, we recently found that temporal upregulation of *Nix*, but not other mitophagy molecules such as *Bnip3, Pink1*, or *Parkin*, occurs during effector memory formation in virus-specific CD8^+^ T cells (18). This upregulation of *Nix* expression leads to clearance of dysfunctional mitochondria selectively during effector memory, but not central memory formation (18). These results suggest that CD8^+^ T cells use autophagy to prevent accumulation of dysfunctional mitochondria during the contraction phase to sustain optimal long-chain fatty acid oxidation during memory formation. This also explains why increasing autophagy via rapamycin and spermidine treatment enhances memory formation in virus-specific CD8^+^ T cells (16, 80).

In spite of these advances in the field, there are some emerging questions that still stand out. Although lysosomal lipolysis and autophagy are known to independently play a role in regulating CD8^+^ T cell memory formation (17, 18, 79, 80), whether the two pathways overlap is still unknown. Even if they are interdependent, it's still unclear as to how cellular lipids are selectively targeted by autophagosomes to be delivered to lysosomes; given the fact that research in the past decade increasingly suggest autophagy being a selective process in contrast to what was thought earlier (91). Furthermore, roles played by other mitophagy-mediating molecules such as FUNDC1 and BCL2L13, which are also outer mitochondrial membrane proteins that mediate mitophagy in non-immune cells (92, 93), are still unknown in the context of T cell memory formation during acute viral infections. Even though NIX is critical for the formation of T cell memory against acute viral infections, it will be interesting to determine whether NIX overexpression during the contraction phase would promote T cell memory formation against latent viral infections.

### Regulation of Short/Branched-Chain Fatty Acid Metabolism During T Cell Memory Formation

In contrast to long-chain fatty acids, short/branched-chain fatty acids are synthesized *de novo* from branched-chain amino acids by T cells (18, 94, 95); and their supply is upregulated under conditions where the availability of long-chain fatty acids becomes limited such as absence of NIX during the contraction phase (18). Short/branched-chain fatty acid metabolism involves ß-oxidation of 2-methylbutyrate, isobutyrate and isovalerate by short/branched-chain specific acyl-CoA dehydrogenase (ACADSB) inside mitochondria to generate ATP molecules (Figure 2B). We recently reported that short/branched-chain fatty acid oxidation plays a critical role in immunological memory formation in virus-specific CD8^+^ T cells in mice (18). Synthesis of short/branched-chain fatty acids is regulated by branched-chain-α-keto acid dehydrogenase kinase (BCKDK) (94), an enzyme that inhibits the flux of short/branched-chain fatty acid oxidation during T cell memory formation (18). We further showed that BCKDK expression is negatively regulated by HIF1α; and accumulation of HIF1α during the contraction phase results in reduced BCKDK expression, thereby increasing the flux of short/branched-chain fatty acid oxidation during anti-viral T cell memory formation (18).

Short/branched-chain fatty acid oxidation generates less ATP than long-chain fatty acid oxidation during memory formation in virus-specific CD8^+^ T cells; and is hence bioenergetically less efficient (18). Upon deletion of NIX in virus-specific CD8^+^ T cells, reduction in long-chain fatty acid oxidation during viral infection is accompanied by an upregulation of short/branched-chain fatty acid oxidation to partially compensate for the loss in ATP synthesis (18). It is still unclear why short/branched-chain fatty acid oxidation is selectively upregulated despite the availability of bioenergetically more efficient options such as medium and very long chain fatty acids. One possible explanation could be that medium-chain fatty acids have sterically bulkier carbon chain which may restrict their diffusion into the mitochondria unlike short/branched-chain fatty acids. Very long chain fatty acids, on the other hand, require ATP-dependent active transporters- a situation which may further deplete ATP during the contraction phase wherein availability of optimal ATP is critical for virus-specific MPECs to successfully differentiate into memory CD8^+^ T cells (18, 96). These hypotheses, however, will need to be experimentally tested. In addition, the molecular mechanism underlying negative regulation of BCKDK expression by HIF1α is still unknown; and it will be interesting to study whether HIF1α can directly repress the transcription of the *Bckdk* gene.

### Epigenetic Regulation of T Cell Memory Formation and Metabolism

Although the current understanding of the molecular mechanisms driving the expression of various genes during T cell memory formation is limited, analysis of epigenetic landscape shows that during T cell memory formation, trimethylation on H3K27 sites (transcriptionally restrictive) reduces on *Atg7* (97), an essential gene for autophagy extensively studied in lymphocytes (79, 80, 98). The corresponding increase in autophagy also corelates with a decrease in the repressive H3K27 trimethylation around *Cpt1a* gene during memory formation (97). This likely results in a more open chromatin structure around autophagy and long-chain fatty acid oxidation genes that could explain increased dependence on fatty acid metabolism during CD8^+^ T cell memory formation (Figure 2C). In the same study, H3K27 trimethylation around *Slc32a1* gene, which mediates glutaminolysis in activated CD8^+^ T cells, was shown to increase in memory CD8^+^ T cells. This suggests that in conjunction with an increase in fatty acid oxidation, effector CD8^+^ T cells make glutaminolytic genes less accessible during differentiation into memory CD8^+^ T cells; thereby funneling available metabolic intermediates toward fatty acid oxidation rather than cellular biogenesis that is otherwise typical of T cell activation. In addition, SIRT1-mediated activation of autophagy is critical for maintaining optimal mitochondrial quality in mammalian tissues (99), suggesting that deacetylation of autophagy genes may also play an important role in positively regulating mitochondrial metabolism. However, whether this role of SIRT1 also applies to differentiation of memory CD8^+^ T cells needs to be experimentally verified through future studies. In addition, RUNX3, a RUNX2 paralog (100), has been shown to increase chromatin accessibility during T cell memory formation; thereby promoting the differentiation of virus-specific memory CD8^+^ T cells (101). Although the exact epigenetic mechanism through which RUNX3 increases the chromatin accessibility during T cell memory formation is still not clear, it has been reported that *Runx3* itself is increasingly demethylated in virus-specific memory precursor CD8^+^ T cells presumably via downregulation of DNA methyltransferase DNMT3a (68, 102–104).

Depending on the stage of T cell differentiation, a transcription factor could induce different metabolic fates. For example, HIF1α upregulates glycolytic genes (as described above) during CD8^+^ T cell activation (10) but activates short/branched-chain fatty acid metabolism during CD8^+^ T cell memory formation (18). The molecular mechanism regulating transcription factor's ability to induce different metabolic pathways at different stages of T cell differentiation is still unknown. However, it's possible that the changing epigenetic landscape during T cell memory formation increases the accessibility of new genes to these transcription factors. Consistent with this possibility, trimethylation of H3K27, deacetylation and demethylation around genes regulating short/branched-chain fatty acid oxidation could play a role here, but this needs to be experimentally demonstrated. Furthermore, the epigenetic mechanism behind contrasting regulation of HIF1α protein level during T cell activation vs memory formation needs to be further elucidated. Although it has been shown that methylation of HIF1α protein by methyltransferases G9a and GLP prevents HIF1α protein accumulation in neuroblastoma cells (71), it is still unknown whether HIF1α downregulation during the contraction phase is also mediated via increased G9a/GLP-dependent HIF1α protein methylation. Further studies are necessary to understand the complete epigenetic story underlying the proclivity of virus-specific memory CD8^+^ T cells to choose long- and short/branched-chain fatty acid oxidation.

## Concluding Remarks

The choice of metabolic pathway in naïve, effector and memory CD8^+^ T cells depends very much on their state of differentiation and functions. Since naïve CD8^+^ T cells are less differentiated compared to their successor T cell repertoires, their ultimate objective is long-term survival so as to be available for differentiation into activated effector T cells when necessary. Mere survival requires basal levels of metabolism, which results in a low demand for energy. This is likely responsible for choosing basal levels of glycolysis and fatty acid oxidation in naïve CD8^+^ T cells. On the other hand, effector CD8^+^ T cells must not only proliferate but also produce exponentially higher level of ATP to feed the process of clonal proliferation and effector functions by funneling energy into a myriad of enzymatic processes occurring during T cell activation. This results in upregulated glycolysis and glutaminolysis to produce biosynthetic intermediates needed for cellular proliferation and meet the increasing energy demands. Since the main function of memory CD8^+^ T cells is long-term survival in order to protect against future re-infection, their quiescent metabolism is driven by autophagy-mediated fatty acid oxidation. Autophagy may serve two purposes: to extract fatty acids from cellular lipid stores for fatty acid oxidation; and degrade dysfunctional organelles so that the memory T cells can replace them with quality ones in order to survive long-term and re-activate optimally upon re-encountering pathogens.

## Author Contributions

SG wrote the manuscript. MC and JW revised the manuscript.

## Conflict of Interest

The authors declare that the research was conducted in the absence of any commercial or financial relationships that could be construed as a potential conflict of interest.
